# Egg Production Constrains Chemical Defenses in a Neotropical Arachnid

**DOI:** 10.1371/journal.pone.0134908

**Published:** 2015-09-02

**Authors:** Taís M. Nazareth, Glauco Machado

**Affiliations:** 1 Programa de Pós-graduação em Ecologia, Departamento de Ecologia, Instituto de Biociências, Universidade de São Paulo, Rua do Matão, trav. 14, no. 321, São Paulo, SP, 05508–900, Brazil; 2 LAGE do Departamento de Ecologia, Instituto de Biociências, Universidade de São Paulo, Rua do Matão, trav. 14, no. 321, São Paulo, SP, 05508–900, Brazil; CNRS, FRANCE

## Abstract

Female investment in large eggs increases the demand for fatty acids, which are allocated for yolk production. Since the biosynthetic pathway leading to fatty acids uses the same precursors used in the formation of polyketides, allocation trade-offs are expected to emerge. Therefore, egg production should constrain the investment in chemical defenses based on polyketides, such as benzoquinones. We tested this hypothesis using the harvestman *Acutiosoma longipes*, which produces large eggs and releases benzoquinones as chemical defense. We predicted that the amount of secretion released by ovigerous females (OFs) would be smaller than that of non-ovigerous females (NOF). We also conducted a series of bioassays in the field and in the laboratory to test whether egg production renders OFs more vulnerable to predation. OFs produce less secretion than NOFs, which is congruent with the hypothesis that egg production constrains the investment in chemical defenses. Results of the bioassays show that the secretion released by OFs is less effective in deterring potential predators (ants and spiders) than the secretion released by NOFs. In conclusion, females allocate resources to chemical defenses in a way that preserves a primary biological function related to reproduction. However, the trade-off between egg and secretion production makes OFs vulnerable to predators. We suggest that egg production is a critical moment in the life of harvestman females, representing perhaps the highest cost of reproduction in the group.

## Introduction

The production of large and heavily yolked eggs is perhaps the most widespread form of parental care among animals [1]. Egg size is related to survival and growth of early hatched young in multiple taxa, including arthropods [2], fishes [3], amphibians [4], and birds [5]. Despite the benefits to the offspring, the production of large eggs may also impose costs to females because reproduction and self-maintenance are two processes that require great investment of energy and resources (review in [6]). Indeed, a negative correlation between survival and female investment in current reproduction is one of the most ubiquitous life-history trade-offs reported in the literature [7,8]. This pattern may emerge as a consequence of several different processes, but the allocation trade-off between reproduction and immune function has received the most attention in recent years [9,10]. Experimental evidence of insects, lizards, and birds has consistently shown that increases in the reproductive effort lead to decreases in the immune function and vice-versa (e.g., [11–15]).

Although intensively studied, the immune system is one of the last lines of defense against natural enemies exhibited by animals [16]. Comparatively, few studies have investigated possible trade-offs between reproduction and other types of defense; most of them are focused on plants in which an increase in induced chemical defenses against herbivores promotes a decrease in fruit or seed production ([17, 18]; but see [19]). There is also indirect evidence suggesting a trade-off between reproduction and chemical defenses among some marine animals. In the marine bryozoan *Membranipora membranacea*, for instance, colonies rapidly produce defensive spines in response to cues from a specialized predatory gastropod. In this case, colonies producing spines grow at lower rates than control colonies, and this growth decrease is directly translated into a reduced output of sexual propagules because fecundity is positively related to colony size in bryozoans [20]. In the sponge *Oscarella balibaloi*, the production of secondary metabolites decreases during the period of embryogenesis, suggesting a trade-off between the resources dedicated to reproduction and the production of chemical defenses [21].

Given that chemical defenses are extremely common among many taxonomic groups, and that there is strong evidence showing that these chemical defenses are costly (review in [22]), it is surprising that the trade-off between reproductive investment and the production of chemical weaponry has never been directly addressed in animals. Arthropods are perhaps one of the most tractable animal groups to explore this trade-off for several practical reasons: (a) many species are chemically defended [23], (b) the composition and biosynthetic pathways of many chemical compounds are well-known [24], (c) female investment in egg size presents huge variation among taxa [25], and (d) chemical defenses may be costly, and thus may compete for resources and energy with other life-history traits. Experimental evidence indicates that synthesizing chemical defenses can slow larval growth in holometabolous insects and also promote a reduction in the final size of the adults, which in turn may reduce their reproductive success (see [26] and references therein).

**Fig 1:**
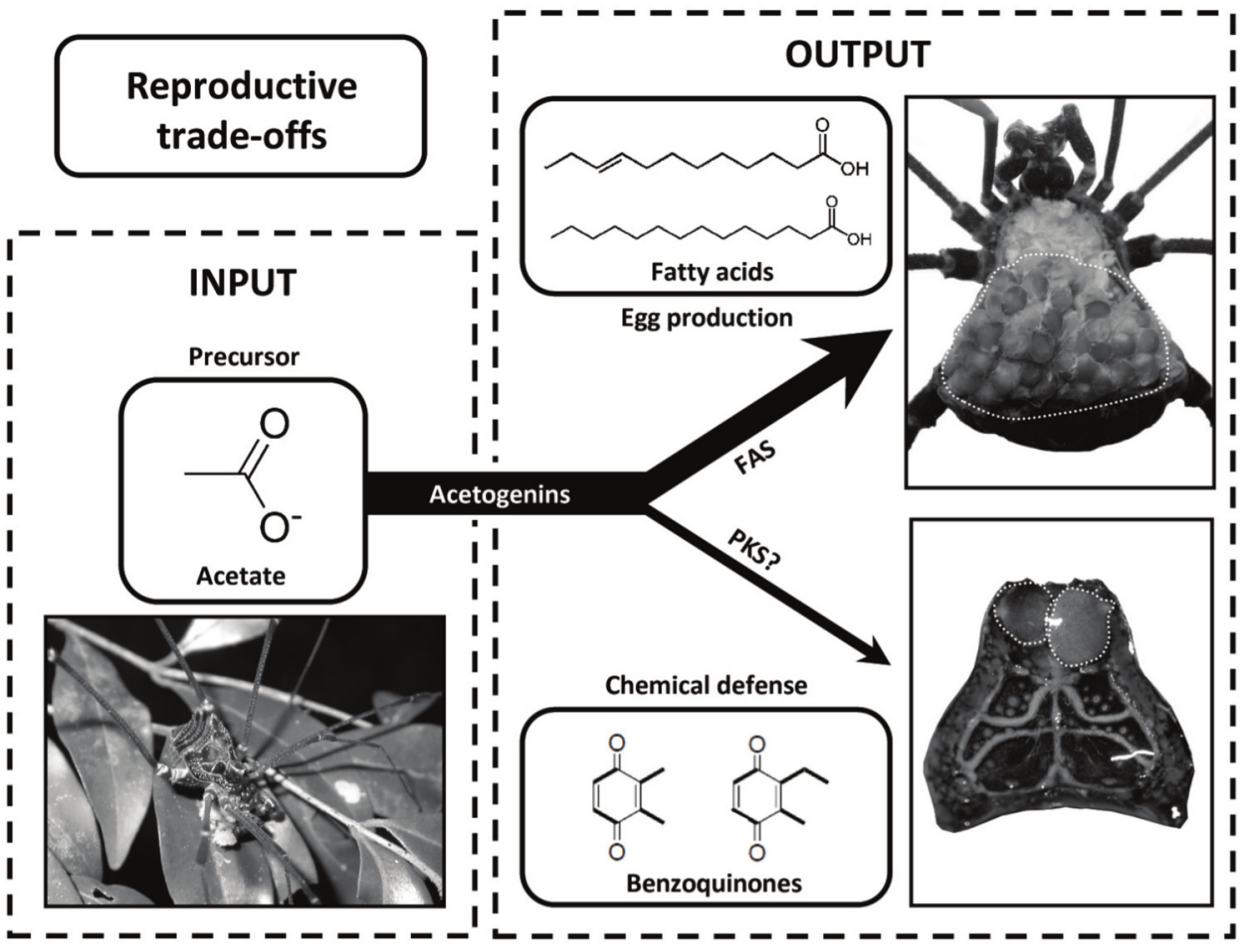
Schematic representation of the of Y model of resource allocation, which proposes that limited resources allocated to reproduction are not available for the rest of the body. In our study, resource input is shown at the left box. Acetate, which is an import precursor of many organic molecules, is acquired when harvestman females feed on live and dead arthropods, fungi, and fruits. The output is shown in the right box illustrating the trade-off between egg production and chemical defenses. The investment in large and heavily yolked eggs in harvestmen (indicated by the white dotted line in the upper photo) increases the demand for fatty acids, which are allocated for the production of the vitelline membrane and lipid droplets imbedded in the yolk. The biosynthetic pathway leading to long chain fatty acids is analogous to the formation of polyketides, and the same precursor (acetate) may be used by both fatty acid synthases (FAS) and polyketide synthases (PKS). Benzoquinones are repellent polyketides produced by many harvestman species in a pair of exocrine glands located at the anterior margins of the carapace (indicated by the white dotted line in the lower photo). As a consequence of allocation trade-offs, the investment in chemical defenses based on benzoquinones should be constrained by egg production.

Quinones are polyketides found in the defensive glands of a wide range of arthropod species, including earwigs, cockroaches, termites, grasshoppers, beetles, millipedes, and harvestmen [23]. Despite the great diversity of quinonoid compounds produced by these arthropods, there are only two metabolic pathways for the generation of benzoquinones [27]. In millipedes (Diplopoda) and insects, benzoquinones may be biosynthesized from preformed aromatic rings of amino acids, such as tyrosine, or using acetate or propionate as precursors, suggesting a polyketide origin [27–29, 24]. In harvestmen (Opiliones), however, alkylated benzoquinones seem to be biosynthesized exclusively using acetate and propionate as precursors [27, 30] (Fig 1). According to the Y model of resource allocation, limited resources allocated to reproduction are not available for the soma [6]. Under the perspective of an arthropod female, the investment in large and heavily yolked eggs increases the demand for fatty acids, which are allocated for the production of the vitelline membrane and lipid droplets imbedded in the yolk [31] (Fig 1). Given that the biosynthetic pathways leading to long chain fatty acids are analogous to the formation of polyketides, and that the same precursors are used by both fatty acid synthases and polyketide synthases, allocation trade-offs are expected to emerge [29] (Fig 1).

In this study, we tested the hypothesis that egg production constrains the investment in chemical defenses based on polyketide compounds, such as alkylated benzoquinones, using the harvestman *Acutiosoma longipes* (Gonyleptidae) as study organism (more details in ‘Study species’ below). Our prediction was that the amount of secretion stored in the glandular sac of ovigerous females would be smaller than the amount stored by non-ovigerous females. We also conducted a series of bioassays in the field and in the laboratory to test whether egg production renders ovigerous females more vulnerable to predation. Our predictions were: (i) the amount of secretion released by ovigerous females would be less effective in deterring potential predators than the amount released by non-ovigerous females, and (ii) the chemical shield provided by the defensive secretion [32] would last longer in the non-ovigerous than in the ovigerous females.

## Methods

### Study Species

Individuals of *A*. *longipes* produce a large amount of defensive secretion composed of two alkylated 1,4-benzoquinones that are released through a pair of exocrine glands located at the anterior margins of the carapace [32] (Fig 1). Although these benzoquinones are highly effective in repelling several invertebrate and vertebrate predators, they are employed only when all other evasive measures were unsuccessful in preventing the predator attack [32, 33], which suggests that their production is costly. Females also produce 80–200 large yolked eggs that occupy more than 50% of their body volume before oviposition [34, 35] (Fig 1). These eggs are laid on rock walls inside caves and are guarded by the mother until hatching and dispersal of the nymphs [34].

### Collection of Individuals

We collected individuals of *A*. *longipes* inside caves at Parque Florestal do Itapetinga (23°15’ S; 46°45’ W), Atibaia, state of São Paulo, southeastern Brazil, between October 2003 and May 2004 (COTEC permission #41.852/2001). We selected adult females in three phases of their reproductive cycle. (1) Non-ovigerous females (NOFs) were those bearing no egg and that were not guarding eggs. (2) Ovigerous females (OFs) were those bearing mature eggs and that were about to oviposit (Fig 1). These females can be easily recognized because they show free tergites spaced out with the intersegmental membrane clearly visible [35]. (3) Egg-guarding females were those that already oviposited and were guarding their eggs for nearly 15 days. The age of a clutch in harvestman can be easily inferred because eggs change in color and size over the course of the embryonic development [36]. We selected 15 day-old clutches because previous laboratory experiments with other quinone-releasing harvestman species indicate that it is the time requested for starved individuals to recover most of their gland volume (Nazareth et al. unpublished data). In the laboratory, we placed females belonging to each reproductive phase in different terraria (60 x 40 cm base, 35 cm high) containing pieces of cotton wetted with water to maintain the humidity.

To exclude the possibility that the concentration of the secretion differs between females in different reproductive phases, we sampled additional ovigerous and non-ovigerous females in the same locality in May 2014. We did not sample egg-guarding females because our previous collection indicated that there is no difference in the mass of secretion produced by females in this phase and non-ovigerous females (see Results).

### Production of Chemical Defenses

We quantified the mass of defensive secretion released by females in each reproductive phase 24 h after collection in the field. First, we weighed a small piece of cotton wool, seized an individual by hand, and induced the emission of exudate by pressing the cotton wool held by tweezers against the gland openings. We repeated this procedure three times to ensure that the gland sacs were completely depleted, and then weighed the cotton wool again. Since harvestmen usually release water from the mouth before or after the emission of defensive secretion [36], we blocked the mouthparts of the females with another piece of cotton wool when milking them of secretion to avoid that enteric water fluid would mix with the gland exudate. We discarded this piece of cotton wool soaked with water, and used the difference in weigh between the second and the first measurements of the cotton wool soaked with exudate to estimate the mass of secretion released by each female. Finally, we measured the dorsal scute length (DSL) of each female using digital calipers (to nearest 0.01 mm). DSL is a standard estimate of body size in harvestmen because it does not change according to hunger or reproductive phase [37].

To quantify the concentration of benzoquinones released by OFs and NOFs from the second sample, we induced the emission of exudate as described above. We then washed the cotton wool tree times with 500 μl of CH_2_Cl_2_ to guarantee complete extraction of benzoquinones. We added 1 μl of the obtained solution (solvent and secretion) to 600 ul of CH_2_Cl_2_ and 1 μl of benzophenona (internal standard), and analyzed the sample by gas chromatography. We used a Shimadzu GC-FID 2014 gas chromatograph coupled with an AOC20i autosampler fitted with a RTX-5 capillary column. The oven temperature was as follow: 40°C (2 min), 5°C min^-1^ to 200°C (4 min). Nitrogen was used as the carrier gas at a linear velocity, column flow, and purge of 18.7 cm s^-1^, 1 mL min^-1^ and 3 mL min^-1^, respectively. Injections of 1 uL were carried out in a splitless mode, during 1 min at 220°C and 32.5 kPa. Temperature, air (20% O_2_ in N_2_), and hydrogen flows of detector were set at 250°C, 400 and 40 ml min^-1^, respectively. We calculated the relative amount of the two benzoquinones contained in each sample by the ratio between the area and mass of the internal standard and the area of the benzoquinones in each sample. We estimated total benzoquinones per sample as the sum of the net quantities of the two benzoquinones present in the mixture released by *A*. *longipes* females. We quantified benzoquinones from the linear regression equation (R^2^ = 0.994) of a calibration curve constructed for 1,4-benzoquinone, so that all amounts are expressed as 1,4- benzoquinone equivalents.

We used general linear models (GLMs) to compare the mass of secretion and concentration of benzoquinones released by females (response variables with Gaussian error distribution) in different reproductive phases (categorical predictor variable), controlling for the effect of body size (continuous predictor variable). Given that we did not expect any interaction between reproductive phase and body size, our models include only the additive effect of these variables (exploratory analyses showed that the interaction is indeed not significant; data not shown).

### Bioassays

We conducted a series of bioassays to test the efficiency of NOF and OF secretions against two groups of potential predators: ants and spiders. Immediately before each trial, we milked a female of *A*. *longipes* of secretion by seizing it by hand and collecting the exudate with capillary tubes. We never repeated the same female in different trials because repeated milking of the same individual reduces the amount of released secretion.

### Tests with Ants

We conducted two bioassays to test the potential effect of the secretion on ants: one in the field to test the repellent potential of NOF and OF secretions, and another in the laboratory to test the effectiveness of NOF and OF secretions as a chemical shield. The field experiment consisted of presenting baits made up of pieces of filter paper (1 cm^2^) embedded with a saturated sugar solution and placed on plastic dishes (5 cm diameter). We randomly distributed 100 baits on the forest floor (5 m from each other) at Parque Florestal do Itapetinga. When ants were feeding at the margin of the bait, we stimulated them discharging a solution in the center of the filter paper with a syringe. The constitution of this solution differed among three experimental groups: (1) glandular secretion of one NOF diluted in 100 μl of water, (2) glandular secretion of one OF diluted in 100 μl of water, and (3) 100 μl of distilled water (control). In the three experimental groups, we counted the number of ants in contact with the bait before and 5 s after presentation of solution.

To analyze the data, we created a repellency index (*RI*) given by *RI = (N*_*0*_ *–N*_*5*_ *)/N*_*0*_, where *N*_*0*_ is the number of ants in contact with the bait before and *N*_*5*_ is the number of ants 5 s after presentation of solution. The *RI* indicates the proportion of ants that were repelled from the sugar bait after stimulation, so that when *RI* = 0 no ant was repelled and when *RI* = 1 all ants were repelled from the bait. We performed a GLM on the repellency index (response variable with Gaussian error distribution) including ant species and the experimental groups as predictor categorical variables. Given that the response of the workers to the different experimental groups could vary according to the ant species, we included the interaction between these two variables in the model. If egg production constrains the production of chemical defenses, OF secretion should be less efficient in repelling ants. Thus, the reduction in the number of ants in contact with the baits after stimulation with OF secretion should be intermediate between the control group and those stimulated with NOF secretion.

In the laboratory experiment, we used five colonies of large predatory ants that occur synoptically with *A*. *longipes*: two colonies of *Odontomachus chelifer* and three of *Pachycondyla striata* (both Ponerinae). We collected all colonies in the field and brought them to the laboratory, where they were placed inside plastic trays (25 x 40 cm). In each tray, we placed two test tubes (2 cm diameter x 15 cm length) containing water trapped behind a cotton plug that were used as nests by the ants. Nearly two months after the colonies were brought to the laboratory, we conducted the experiment of chemical shield. Inside each tray, we presented a glass cover slip (1 x 10 cm) divided in three equal parts randomly designated as treatment 1, treatment 2, and control. Treatments 1 and 2 consisted of a filter paper (1 cm^2^) wetted with 100 μl of a saturated sugar solution mixed with the glandular secretion of one NOF and one OF, respectively. The control contained only a filter paper (1 cm^2^) wetted with 100 μl of a saturated sugar solution. We counted the total number of ants feeding on each bait at 2 min-intervals during 40 min after the first contact.

To analyze the data, we performed repeated measures analysis of variance using the number of ants feeding on the baits at each 2 min-interval as response variable and experimental groups as predictors. Degrees of freedom were corrected using the Greenhouse-Geisser procedure to avoid sphericity problems. Given that the number of colonies of each ant species was limited, we did not include species identity in the analysis. If egg production constrains the production of chemical defenses, the chemical shield promoted by OF secretion should last less time than that promoted by NOF secretion for both ant species.

### Test with Spiders

*Trechaleoides biocellatus* (Trechaleidae) is a large (2–3 cm body length) wandering spider, abundant in the study site, which is generally found near river margins or in other moist habitats, such as caves [32]. We collected individuals of the species at Parque Florestal do Itapetinga between October 2003 and May 2004, and maintained them in individual cages (20 x 10 cm base, 15 cm high) containing a piece of cotton wetted with water to maintain the humidity. Only subadults and adults of both sexes (*n* = 60) were used in the experiments, and each individual was starved for 5–6 days before the experiments to bring them to a similar level of hunger.

To test the role of the defensive secretion alone and exclude the interference of other possible defenses, such as spines on legs and pedipalps, we did not offer individuals of *A*. *longipes* directly to the spiders (following [32]). Rather, we offered individuals of the common cricket *Gryllus gryllus* (nearly 1 cm of body length), which the spiders promptly took as prey. In order to ensure that the crickets were unable to promote injuries to the spiders, we removed the hind legs (armed with several spines) of the crickets just before the experiment. After the cricket was grabbed, we stimulated each spider with one of the following solutions: 1) OF secretion diluted in 100 μl of water (*n* = 20); 2) NOF secretion diluted in 100 μl of water (*n* = 20), or 3) 100 μl of distilled water (control). We applied the solutions with a syringe directly to the base of the chelicerae. Spiders that extricated the chelicerae and abandoned the prey within 5 min were scored as respondents (following [38]). We compared the number of spiders that released or not the prey using two Fisher exact tests: one between control and OF secretion, and other between NOF and OF secretions. Given that we performed two analyses using the same dataset, we used the Bonferroni correction to adjust the p values. If egg production constrains the production of chemical defenses, OF secretion should be less efficient in repelling spiders. Thus, the number of crickets released by the spiders stimulated with OF secretion should be lower when compared to the spiders stimulated with NOF secretion.

## Results

### Production of Chemical Defenses

**Fig 2:**
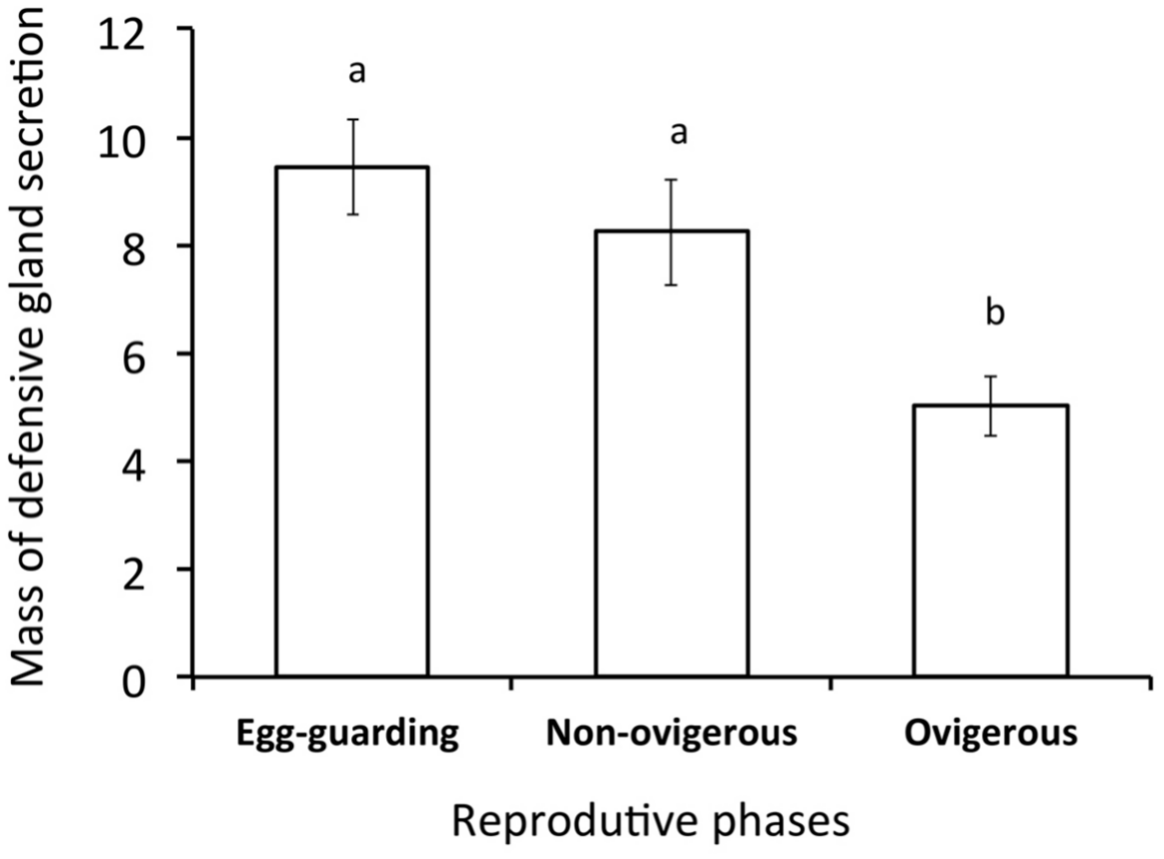
Mass of defensive secretion (mean ± SE) released by egg-guarding, non-ovigerous, and ovigerous females of the harvestman *Acutisoma longipes*. Different letters indicate significant differences (post-hoc test, p < 0.05).

The mass of defensive secretion released by the females was affected by their reproductive phase, but not by their body size. Egg-guarding females (*n* = 12) and NOFs (*n* = 25) released a similar mass of secretion, which was on average 71.8% greater than the mass of secretion released by OFs (*n* = 25; Fig 2; Table 1). Females produced defensive secretion with similar concentration of benzoquinones, regardless of body size and reproductive stage (mean ± SD): NOFs = 21.03 ± 15.28 μg/mL and OFs = 18.97 ± 12.93 μg/mL (Table 1).

**Table 1:** Results of the GLM testing the effect of body size and reproductive phase on the mass of defensive gland secretion and concentration of total benzoquinones released by females of the harvestman *Acutisoma longipes*. Significant p-values are shown in bold.

Effect	DF	MS	F	p
**Mass of defensive secretion**				
Body size	1	35.507	2.430	0.125
Reproductive phase (egg-guarding, non-ovigerous, and ovigerous females)	2	93.733	6.416	**0.003**
Error	58	14.611		
**Concentration of total benzoquinones**				
Body size	1	0.040	0.315	0.576
Reproductive phase (non-ovigerous and ovigerous females)	1	0.168	1.296	0.258
Error	83	0.128		

### Tests with Ants

**Fig 3:**
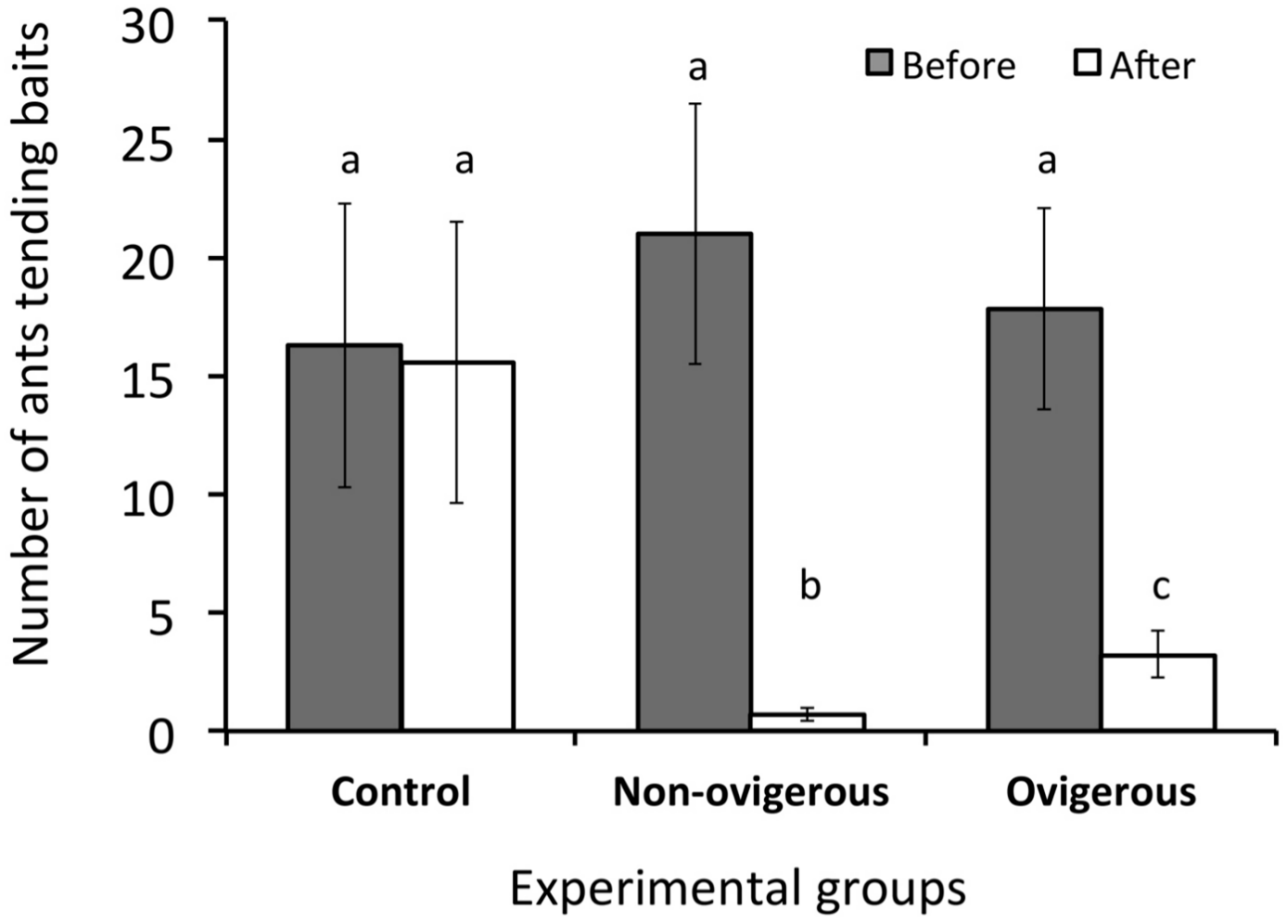
Results of the field experiment in which the number of ants tending sugar baits was counted before and after stimulation with one of the three experimental groups: (1) secretion of one non-ovigerous female of *Acutisoma longipes*, (2) secretion of one ovigerous female of *A*. *longipes*, and (3) distilled water (control). Different letters indicate significant differences (post-hoc test, p < 0.05).

In total, 51 baits were visited by workers of seven ant species in the field: three by *Crematogaster* sp. (one for each experimental group), three by *Pachycondyla striata* (one for each experimental group), three by *Pheidole* sp.1 (one for each experimental group), six by *Odontomachus chelifer* (two for each experimental group), nine by *Camponotus* sp. (three for each experimental group), nine by *Pheidole* sp.2 (three for each experimental group), and 18 by *Gnamptogenys* sp. (six for each experimental group). The discharge of defensive secretions induced a marked reduction in the number of ants feeding on the sugar baits, regardless of the ant species (Table 2). Baits treated with NOF secretion, however, had slightly higher repellency than those treated with OF secretion (Fig 3). No significant reduction was observed in the control baits (Fig 3).

**Table 2:** Results of the GLM testing the effect of ant species and experimental groups on the repellency index. The experimental groups were: (1) secretion of one non-ovigerous female of *Acutisoma longipes*, (2) secretion of one ovigerous female of *A*. *longipes*, and (3) distilled water (control). Significant p-values are shown in bold.

Effect	DF	MS	F	p
Intercept	1	13.109	601.975	**< 0.001**
Ant species	6	0.013	0.609	0.721
Experimental group	2	2.664	122.322	**< 0.001**
Ant species x Experimental group	12	0.014	0.661	0.773
Error	30	0.022		

**Fig 4:**
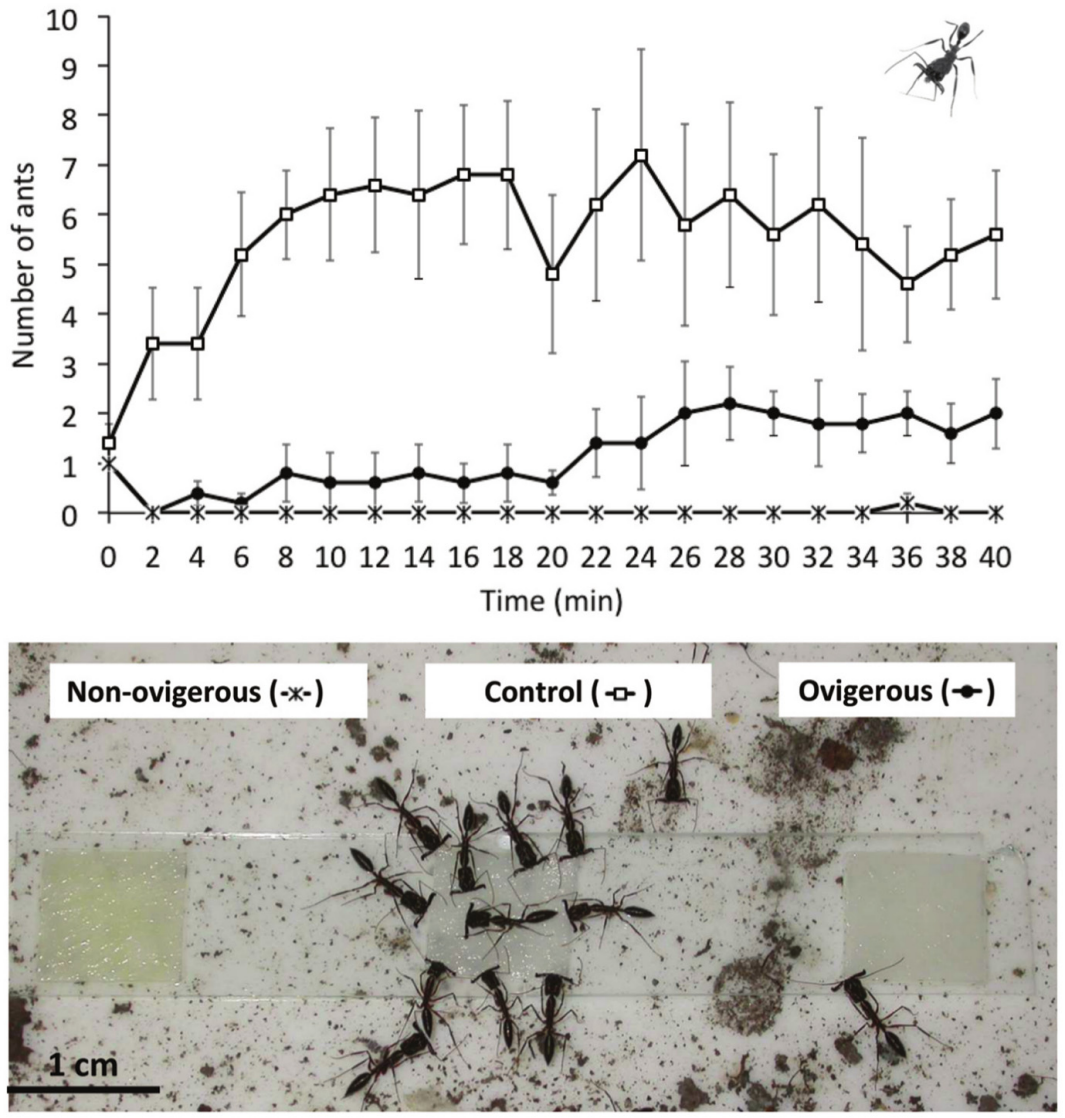
Mean (± SE) number of workers of the ants *Odontomachus chelifer* and *Pachycondyla striata* tending three types of baits: (1) sugar solution + secretion of one non-ovigerous female of *Acutisoma longipes*, (2) sugar solution + secretion of one ovigerous female of *A*. *longipes*. and (3) sugar solution (control). The photo illustrates the experimental setup of an *O*. *chelifer* colony at 14 min of experiment.

In the laboratory bioassay, the number of workers tending control baits increased fast from 0 to 12 min. After this period until the end of the experiment, we observed, on average, five to seven workers tending the baits (Fig 4). No ant was observed tending the baits containing OF secretion during the first 2 min. After 4 min, however, the number of workers tending the baits increased slowly, and from 22 min until the end of the experiment, we observed, on average, one to two workers tending the baits (Fig 4). No ant was observed tending the baits containing NOF secretion during the entire experiment in almost all colonies (Fig 4). In general, there was a significant interaction between time and experimental group (*F*_*40*, *160*_ = 4.55; p = 0.002).

### Tests with Spiders

Only one individual of *T*. *biocellatus* released the prey in the control group (*n* = 20). When individuals were stimulated with OF secretion (*n* = 20), only two released the prey and this frequency does not differ from the control (Fisher exact test, p = 1.00). When individuals were stimulated with NOF secretion (*n* = 20), nine released the prey and this frequency is significantly higher than the group stimulated with OF secretion (Fisher exact test, p = 0.016).

## Discussion

We found that females of the harvestman *A*. *longipes* bearing mature eggs produce less defensive secretion than females in other reproductive phases, including non-ovigerous and egg-guarding females. Moreover, we found no difference in the concentration of total benzoquinones released by OFs and NOFs, indicating that there is no compensation related to increased concentration of defensive compounds during egg production. These results are congruent with the hypothesis that egg production constrains the investment in chemical defenses based on benzoquinones (Fig 1). Finally, the results of our bioassays clearly indicate that the low amount of secretion released by OFs is less effective in deterring potential predators (ants and spiders) than the high amount released by NOFs. In what follows, we will explore these results in more detail and discuss their implications for our understanding on the costs of reproduction in a chemically defended animal.

During egg production, a large amount of yolk must be stored in the oocytes in a relatively short period of time to provide nutritional supply for the developing embryo [39]. The increased physiological requirement promoted by egg production leads females of many species to intensify their foraging activities [40]. In the fishing spider *Dolomedes triton*, for instance, females switch from a sit-and-wait strategy to more active foraging upon maturation [41,42]. In the harvestman *Serracutisoma spelaeum*, OFs forage more frequently than NOFs, leaving the cave habitat to search for food almost every night [42]. The same pattern seems to occur with *A*. *longipes* (G. Machado, pers. obs.), and our results suggest that the resources acquired by OFs during this period of intense foraging activity are invested predominantly in egg production, rather than chemical defenses. Histological studies of *A*. *longipes* support this suggestion, indicating that during the period of egg production there is a marked increase in the lipid content of the fat body [44], an organ that is the source of most part of the yolk received by the ovarian follicles [31].

The possible trade-off reported here for *A*. *longipes* contrasts with the results obtained in a previous study with *Zophobas atratus*, a tenebrionid beetle that produces two alkylated benzoquinones also found in many harvestman species [30]. In this beetle, egg production did not affect the investment in chemical defenses, so that mated females that produced twice as many eggs as virgin females released nearly the same amount of defensive secretions [45]. The authors suggest that individuals do not channel much energy into the production of defensive secretions, and that a marked trade-off between egg and secretion production should be found in species that invest more energy in chemical defenses [45]. We think, however, that the lack of a trade-off reported for *Z*. *atratus* is related to the high abundance of food provided to the beetles in the laboratory. Trade-offs are more likely to emerge when internal energy reserves are limited [6], and under natural conditions, egg-producing *A*. *longipes* females are probably food limited because most foraging trips outside the cave habitat are unsuccessful [33]. This may explain why we detected a negative influence of egg production on the total mass of secretion produced by OFs.

Egg-guarding females released the same mass of secretion reported for NOFs (Fig 2), indicating that the investment in chemical defenses is resumed after oviposition. Given that females are prevented from foraging while caring for the offspring, and remain stationary on the clutch all day long during the entire period of embryonic development [34], which resources are used to produce benzoquinones? Detailed histological studies of *A*. *longipes* show that some oocytes are reabsorbed during the period of maternal care [44], and we suggest that the nutrients obtained from the oocytes are the source for the production of benzoquinones during the caring period. The possible translocation of resources between the endpoints of the Y model of resource allocation reinforces the notion that the same precursors can be used to produce both fatty acids and polyketides [29], giving rise to the trade-off we are proposing here between egg and benzoquinone production (Fig 1). The increased production of chemical defenses after oviposition may be particularly important for egg-guarding females, which remain on the clutch for more than one month probably exposed to active-hunting predators [34]. In fact, a long-term field experiment with the harvestman *S*. *proximum*, which is closely related to *A*. *longipes*, indicates that the mortality of egg-guarding females is not reduced when compared with females prevented from caring [46], suggesting that females are well-protected against predation during the period of parental care.

The strong irritating properties of benzoquinones are known to repel numerous invertebrate and vertebrate predators (see [23] and references therein). In a previous study with *A*. *longipes*, the defensive secretion of adult males and NOFs repelled seven ant species, two species of large wandering spiders, and one frog species [32]. Using similar protocols, we showed here that the efficiency of the defensive gland secretion released by OFs is reduced when compared with NOFs. In the field experiment with ants, the repellency index of OF secretion was slightly lower than that of NOF (Fig 3). However, the variance in the repellency index of the secretion released by OFs was much higher, with some values overlapping the values of the control group (Fig 3). In the laboratory experiment, the chemical shield promoted by the OF secretion lasted considerably less time than that of NOF secretion (Fig 4). After 40 min, an average of two ants was tending the baits wetted with OF secretion while no ant was tending the baits wetted with NOF secretion (Fig 4). Finally, spiders stimulated with OF secretion released the crickets 4.5 times less frequently than those stimulated with NOF secretion.

Field studies with several arthropod species, including three neotropical harvestmen, indicate that individuals that are more active during the reproductive period are more frequently captured by ambush predators than sedentary individuals [37, 47–50]. As we mentioned above, females of goniosomatine harvestmen increase their foraging activities during the period of egg production and leave the cave habitat on a daily basis, while males and NOFs may remain stationary inside the cave for three or more days [43, 33]. Therefore, highly vagile OFs are probably under a higher risk of predation than other conspecifics, especially if the egg load also decreases female locomotor ability, as it has already been reported for females of many animal groups (review in [51]). Additionally, the results of our bioassays suggest that the trade-off between egg and benzoquinone production makes OFs particularly vulnerable to predation when compared with NOF. Taken together, these findings support the notion that egg production is a critical moment in the life of harvestman females, representing perhaps the highest cost of reproduction, as also suggested for many bird species [52].

In conclusion, females allocate resources to chemical defenses in a way that preserves a primary biological function related to reproduction. As far as we know, this is the first time this trade-off has been directly demonstrated for animals. In the future, mark-recapture studies should be conducted in the field to access whether mortality rates of OFs are higher than NOFs. Moreover, it would be interesting to investigate whether OFs fed *ad libitum* in the laboratory are able to channel more resources to egg production, so that the trade-off observed under field conditions is somehow attenuated. Finally, a metabolomic approach to the trade-off between egg and benzoquinone production could be valuable to characterize the physiological responses of the females at the biochemical level (see [53]).
